# Licorice extract inhibits porcine epidemic diarrhea virus in vitro and in vivo

**DOI:** 10.1099/jgv.0.001964

**Published:** 2024-03-12

**Authors:** Wenfei Bai, Qinghe Zhu, Jun Wang, Limin Jiang, Donghua Guo, Chunqiu Li, Xiaoxu Xing, Dongbo Sun

**Affiliations:** 1 College of Animal Science and Veterinary Medicine, Heilongjiang Bayi Agricultural University, No. 5 Xinfeng Road, Sartu District, Daqing 163319, PR China

**Keywords:** antiviral drugs, licorice extract, porcine epidemic diarrhea virus, swine

## Abstract

Porcine epidemic diarrhea virus (PEDV) causes severe diarrhea and even death in piglets, resulting in significant economic losses to the pig industry. Because of the ongoing mutation of PEDV, there might be variations between the vaccine strain and the prevailing strain, causing the vaccine to not offer full protection against different PEDV variant strains. Therefore, it is necessary to develop anti-PEDV drugs to compensate for vaccines. This study confirmed the anti-PEDV effect of licorice extract (Le) *in vitro* and *in vivo*. Le inhibited PEDV replication in a dose-dependent manner *in vitro*. By exploring the effect of Le on the life cycle of PEDV, we found that Le inhibited the attachment, internalization, and replication stages of the virus. *In vivo*, all five piglets in the PEDV-infected group died within 72 h. In comparison, the Le-treated group had a survival rate of 80 % at the same time, with significant relief of clinical symptoms, pathological damage, and viral loads in the jejunum and ileum. Our results suggested that Le can exert anti-PEDV effects *in vitro* and *in vivo*. Le is effective and inexpensive; therefore it has the potential to be developed as a new anti-PEDV drug.

## Introduction

Porcine epidemic diarrhea (PED) is a highly contagious acute intestinal disease caused by porcine epidemic diarrhea virus (PEDV) [1,2]. The disease has a rapid onset and is accompanied by clinical symptoms such as severe diarrhea, vomiting, and dehydration [3]. Until 2010, the disease was not a worldwide concern due to the use of inactivated or attenuated vaccines. However, the emergence of variant PEDV strains of high virulence in China in 2010 led to mortality rates of infected piglets soaring to 80–100 % [4]. In April 2013, PEDV began to spread rapidly in the USA, resulting in the deaths of more than eight million piglets [5]. PEDV positivity has been reported to range from 50.21–62.1 % in samples collected in South China from 2012 to 2018 [6]. In another study, samples of piglet diarrhea were collected from eight regions in China (2011–2021), and PEDV was found to be the primary pathogen, with positive rates consistently exceeding 40 % [3]. These studies have shown that PEDV is still widely spread in Chinese swine herds to this day, causing considerable losses to the pig farming industry. Therefore, the prevention and control of this disease are essential.

The most promising and effective strategy to protect piglets from PEDV is to obtain adequate maternal antibodies from colostrum and milk [7]. Because of the ongoing mutation of PEDV, there might be variations between the vaccine strain and the prevailing strain, causing the vaccine to not offer full protection against different PEDV variant strains [8]. Thus, it is necessary to find new strategies to compensate for vaccines while continuing to develop vaccines based on prevalent strains of PEDV. Researchers have widely emphasized the study of anti-PEDV drugs, and a variety of drugs or natural products with anti-PEDV activity have been screened [1]. Drugs that have been reported to have anti-PEDV effects are dominated by natural products of plant origin. Among the polyphenols, epigallocatechin-3-gallate is a catechin monomer isolated from tea, which exerts antiviral effects by hindering the attachment, internalization, replication, and release of PEDV [9]. Tomatidine is an alkaloid that targets PEDV 3CLpro [10]. Quercetin is a flavonoid molecule that also targets PEDV 3CLpro, and its analogue quercetin 7-rhamnoside affects the initial stages of PEDV infection [11,12]. Four polysaccharides were identified in the aqueous extract of Pogostemon cablin, and all four polysaccharides were found to inhibit PEDV replication [13]. Aloe and chestnut inner shell extracts inhibit PEDV replication [14,15]. Our team showed that octyl gallate can exert anti-PEDV effects *in vitro* and *in vivo* by targeting 3CLpro and has potential broad-spectrum antiviral activity against other porcine enteric coronaviruses [2].

Using plant sources as a starting point for drug development often has the following advantages. Usually, drug candidates for treating diseases can be selected based on human ethnomedicine, including traditional Chinese medicine (TCM) [16]. This approach is based on the assumption that active compounds isolated from these plants may be safer than compounds derived from plant species with no history of human use [17]. Moreover, TCM extracts have a wide range of pharmacological effects, such as anti-inflammatory, antioxidant, and immunomodulatory, which exert indirect antiviral effects [18,19]. However, new drug development is a complex, time-consuming, and expensive process [17]. Some discovered natural products are limited in their use in practice because of the complexity of their extraction processes and high manufacturing costs [20,21]. Another problem is the effectiveness of antiviral drug therapy. Most of the anti-PEDV drugs that have been reported have only been validated for antiviral effects *in vitro*, and their effects in piglets need to be confirmed. Therefore, it is essential to find cheap and effective anti-PEDV drugs.

Licorice is a well-known TCM that was recorded around 2100 BC [22]. For the next several thousand years, licorice was present in most traditional Chinese prescriptions [23]. Previous studies have revealed many pharmacological activities of licorice, such as antiviral [24,25], antibacterial [26], anti-inflammatory [27], and antitumor [28]. Licorice has been reported to inhibit the attachment and internalization of human respiratory syncytial virus [25] and enterovirus 71 [29], reduce transport to the membrane and sialylation of hepatitis B virus surface antigen [30], inhibit the fusion of human immunodeficiency virus to cell membranes [31], and reduce RANTES levels after influenza A virus (H1N1) infection [32]. Licorice is found in traditional anti-diarrheal formulas such as Sijunzi soup, Fuzi lizhong pills, and Renshen jianpi pills [33]. Licorice is inexpensive and readily available and has the potential to be developed into animal medicine [34,35]. In addition, the active ingredients of licorice have potential effects against SARS-CoV2 and related viruses. Since both SARS-CoV2 and PEDV are members of the family *Coronaviridae* and the target proteins share a high degree of homology, licorice has the potential to be anti-PEDV [24]. Meanwhile, glycyrrhizin contained in licorice can inhibit the infection of PEDV and weaken the pro-inflammatory response by inhibiting high mobility group box-1 protein [36]. Therefore, we chose to validate the anti-PEDV effect of licorice extract (Le).

In this study, we extracted licorice and confirmed the anti-PEDV effect of Le *in vitro* and *in vivo*. We showed that Le inhibited PEDV replication *in vitro* and acted on the attachment, internalization, and replication phases of the PEDV life cycle. Subsequently, at safe concentrations, we found that Le inhibited PEDV infection *in vivo*. These findings could provide a basis for the development of inexpensive and efficient anti-PEDV drugs.

## Methods

### Preparation of Le

The licorice (origin: Gansu Province) was dried at 40 °C for 2 days. The dried licorice tablets were pulverized with a shredder, and passed through a fine sieve of 300 mesh. The licorice powder was soaked in 70 % ethanol at room temperature for 3 h, and then the licorice powder was extracted at 70 °C. The extraction process was repeated twice. The extracts were combined and then concentrated using a rotary evaporator at 40 °C and lyophilized using a freeze dryer (Yetuo, China) to obtain the Le.

### Cells and viruses

Vero E6 cells were cultured in Dulbecco’s Modified Eagle’s medium (DMEM) supplemented with 10 % fetal bovine serum and placed in a humidified environment at 37 °C with 5 % CO_2_. The PEDV HM2017 strain (GenBank accession no. MK690502) was maintained in our laboratory [37].

### Cell viability assay

The impact of Le on the activity of Vero E6 cells was assessed utilizing a commercial Cell Counting Kit-8 (CCK-8) (BS350B, Biosharp, Beijing, China). Vero E6 cells in the logarithmic growth phase were transferred to 96-well plates and cultured until they reached 90% confluence. After co-incubation with DMEM or Le (3.91–500 µg) for 48 h, 10 µl of CCK-8 solution was added to each well, and the mixture was incubated for an additional 1 h, protected from light. OD 450 nm was measured using a microplate reader (BioTek Instruments, Winooski, VT, USA), and cell viability was calculated as follows: [OD(drugged) − OD(blank)]/[OD(no drug) − OD(blank)] × 100 %. The 50 % cytotoxic concentration (CC_50_) was analysed with GraphPad Prism 8.0 software.

### Quantitative reverse transcription PCR

Total RNA was extracted from Vero E6 cells using the RNAsimple Total RNA kit (KR118, Tiangen Biotech, China). The cDNA synthesis was performed with random primers using the FastKing gDNA Dispelling RT SuperMix kit (KR116, Tiangen Biotech). Quantitative reverse transcription PCR (qRT-PCR) was performed as described previously [38] using SYBR Green I fluorescent dye (E168, Novoprotein, Suzhou, China) and a QuantStudio three real-time PCR system (Applied Biosystems, Carlsbad, CA, USA). The cDNA was generated by qRT-PCR using specific primers (PEDV ORF3-F: 5′-GCA CTT ATT GGC AGG CTT TGT-3′; PEDV ORF3-R: 5′-CCA TTG AGA AAA GAA AGT GTC GTA G-3′; β-actin-F: 5′-AGG CTC TCT TCC AAC CTT CCT T-3′; β-actin-R: 5′-ACG TCG CAC TTC ATG ATC GA-3′). The qRT-PCR volume was 25 µl, which consisted of 12.5 µl 2×SYBR Premix Ex Taq (TaKaRa Bio, Kusatsu, Japan), 0.5 µl (10 pmol l^−1^) of the forward primer, 0.5 µl (10 pmol l^−1^) reverse primer, 4 µl template DNA, and 7.5 µl sterile water. The reaction was conducted using a protocol consisting of 40 cycles of 95 °C for 30 s, 60 °C for 30 s, and 72 °C for 30 s, followed by a 5 s annealing period at 92 °C and a 1 min annealing period at 60 °C. The reaction was then subjected to an extended period at 95 °C and a cooling period at 50 °C for 30 s. Finally, the Ct values were recorded and analysed.

### Western blotting

Total cellular proteins were extracted using RIPA buffer (R0278; Sigma-Aldrich St. Louis, MO, USA). The extracted proteins were separated using a 10% SDS-PAGE gel and then transferred to polyvinylidene difluoride membranes. The membranes were blocked using 5 % skimmed milk powder and 0.05 % Tween 20 (PBST) in phosphate-buffered saline (PBS) for 2 h. The antibodies against PEDV N protein (1 : 200 dilution) and GAPDH antibody (1 : 1000 dilution) were separately incubated with the membranes at 4 °C overnight. The membranes were treated with a secondary antibody labelled with horseradish peroxidase and were left to incubate for 1 h at room temperature. After washing with 0.05 % PBST, the membranes were exposed to Luminata Crescendo Western HRP substrate (Merck KGaA, Darmstadt, Germany) for detection using an Amersham Imager 600 (GE Healthcare, Chicago, IL, USA). The ImageJ software (National Institutes of Health, Bethesda, MD, USA) was utilized to quantify target protein expression levels.

### TCID_50_ assay

Vero E6 cells were treated with DMEM containing different concentrations of Le (0–60 mg ml^−1^) and then infected with PEDV at MOI 0.2 for 2 h. The supernatants were collected. The viral titre of PEDV in Vero E6 cells was determined according to the 50 % tissue culture infective dose (TCID_50_). Vero E6 cells were inoculated into 96-well plates with 10^5^ cells per well and incubated in 100 µl of DMEM at 37 °C and 5 % CO_2_ for 48 h. The culture medium was removed, and 100 µl of ten-fold serial dilutions of the virus were added to each well. The cytopathic effect was detected every 12 h for 5 days after inoculation. Virus litres were calculated according to the Reed-Muench method [39].

### Immunofluorescence assay

Vero E6 cells were fixed in 4 % paraformaldehyde (BL539A; Biosharp) for 15 min, washed five times with PBS, and PEDV M protein monoclonal antibody (1 : 1000 dilution) was added and incubated overnight at 4 °C. After five washes with PBS, the cells were incubated with fluorescently labelled goat anti-mouse IgG antibody (1 : 2000 dilution) (Merck KGaA, Darmstadt, Germany) for 1 h at room temperature, and stained with DAPI (1 : 250 dilution) (C1005; Beyotime, Shanghai, China) for 15 min. The cells were washed with PBS and observed under a fluorescence microscope.

### Virus inactivation assay

Le (60 µg ml^−1^) or dimethyl sulfoxide (DMSO) was incubated with PEDV (0.01 MOI) at 37 °C for 3 and 5 h. Le (60 µg ml^−1^) or a mixture of DMSO and PEDV was added to a six-well plate lined with Vero E6 cells. After an additional 2 h of incubation at 37 °C, the supernatant in the six-well plate was replaced with fresh virus maintenance solution and incubation was continued for 12 h [10,40]. Cell sample RNA was extracted after completion of the incubation and PEDV ORF3 mRNA was measured using qRT-PCR.

### Virus attachment assay

Vero E6 cells were pretreated with Le (60 µg ml^−1^) or DMSO for 2 h at 37 °C, and then cells were infected with PEDV (0.01 MOI) at 4 °C. After 30 min and 1 h incubation, Vero E6 cells were rinsed with pre-cooled PBS, and then cells were collected [2,10, 40]. The mRNA levels of PEDV ORF3 and β-actin were measured using qRT-PCR.

### Virus internalization assay

Vero E6 cells were infected with PEDV (0.01 MOI) for 2 h at 4 °C. Supernatants were replaced with DMEM containing Le (60 µg ml^−1^) or DMSO and incubated for 1 and 2 h at 37 °C. Cells were washed with citrate buffer (pH 3) to remove the uninternalized virus and collected [2,10, 40]. The mRNA levels of PEDV ORF3 and β-actin were measured using qRT-PCR.

### Virus replication assay

Vero E6 cells were incubated with PEDV (0.01 MOI) for 2 h at 37 °C and washed three times with PBS to remove the free virus. At 4 h post-inoculation (hpi), the supernatant was replaced with fresh virus maintenance solution containing Le (60 µg ml^−1^) or DMSO, and the cells were incubated at 37 °C. Vero E6 cells were washed with PBS at 6, 8, and 10 hpi, respectively, and cells were collected. The mRNA levels of PEDV ORF3 and β-actin were measured using qRT-PCR [2,10, 40].

### Virus release assay

Vero E6 cells were infected with PEDV (0.01 MOI) at 37 °C for 2 h. The medium was then replaced with fresh virus maintenance solution. At 10 hpi, the cells were washed with PBS, and the medium was replaced with virus maintenance solution containing Le (60 µg ml^−1^) or DMSO. The cells were incubated at 37 °C for 0.5, 1, and 2 h, and the supernatant was harvested for detection of mRNA levels of PEDV ORF3 in the released virus by absolute fluorescence quantification [2,10, 40].

### Animal experiments

For Le toxicity assays, 30 BALB/c mice were randomly divided into six groups of five, with each group housed individually. On day 0, mice in group one were gavaged with 0.5 ml DMEM and served as controls. Mice in groups 2–6 were gavaged with 0.5 ml of DMEM containing Le at doses of 4, 20, 100, 500, and 2500 mg kg^−1^, respectively. In acute toxicity studies, murine mortality was observed and recorded for 14 days. For subchronic toxicity assessment, after administration of Le, the body weight of each mouse was measured daily, and blood was collected from all mice 14 days after inoculation for blood cell detection. After deriving the maximum non-toxicity measure, the optimal dose to be administered was calculated based on the relative body surface area of the piglets [41].

In the piglet protection experiment, 15 4-day-old conventional piglets randomly divided into three groups of five and housed in separate rooms: healthy control, PEDV-infected control, and the Le-treated. These piglets tested negative for PEDV, porcine deltacoronavirus, transmissible gastroenteritis virus, and porcine rotavirus using a colloidal gold rapid detection kit. The Le-treated group piglets were orally administered 250 mg kg^−1^ Le every 6 h before feeding. After 1 day, piglets in the Le-treated and PEDV-infected groups were orally administered 3 ml DMEM containing 1×10^4.8^ TCID_50_ ml^−1^ PEDV HM2017. The healthy control group was orally administered an equal amount of DMEM simultaneously. After inoculation, the body temperature and weight of piglets were recorded every 12 h, and clinical symptoms and faecal were scored. After all the infected piglets died, the remaining piglets were euthanized, and small intestinal tissues were collected for histological examination. All animal experiments follow the Guiding Principles for Biomedical Research Involving Animals.

### Statistical analysis

Statistical analysis was conducted utilizing GraphPad Prism eight software. All data were displayed as the average of three or more independent experiments. The results were presented as mean±standard deviation. One-way or two-way analysis of variance was used to assess the significance of any differences between groups (*P<0.05; **P<0.01; ***P<0.001; ns=not significant).

## Results

### Le inhibits PEDV infection *in vitro*

**Fig. 1.**
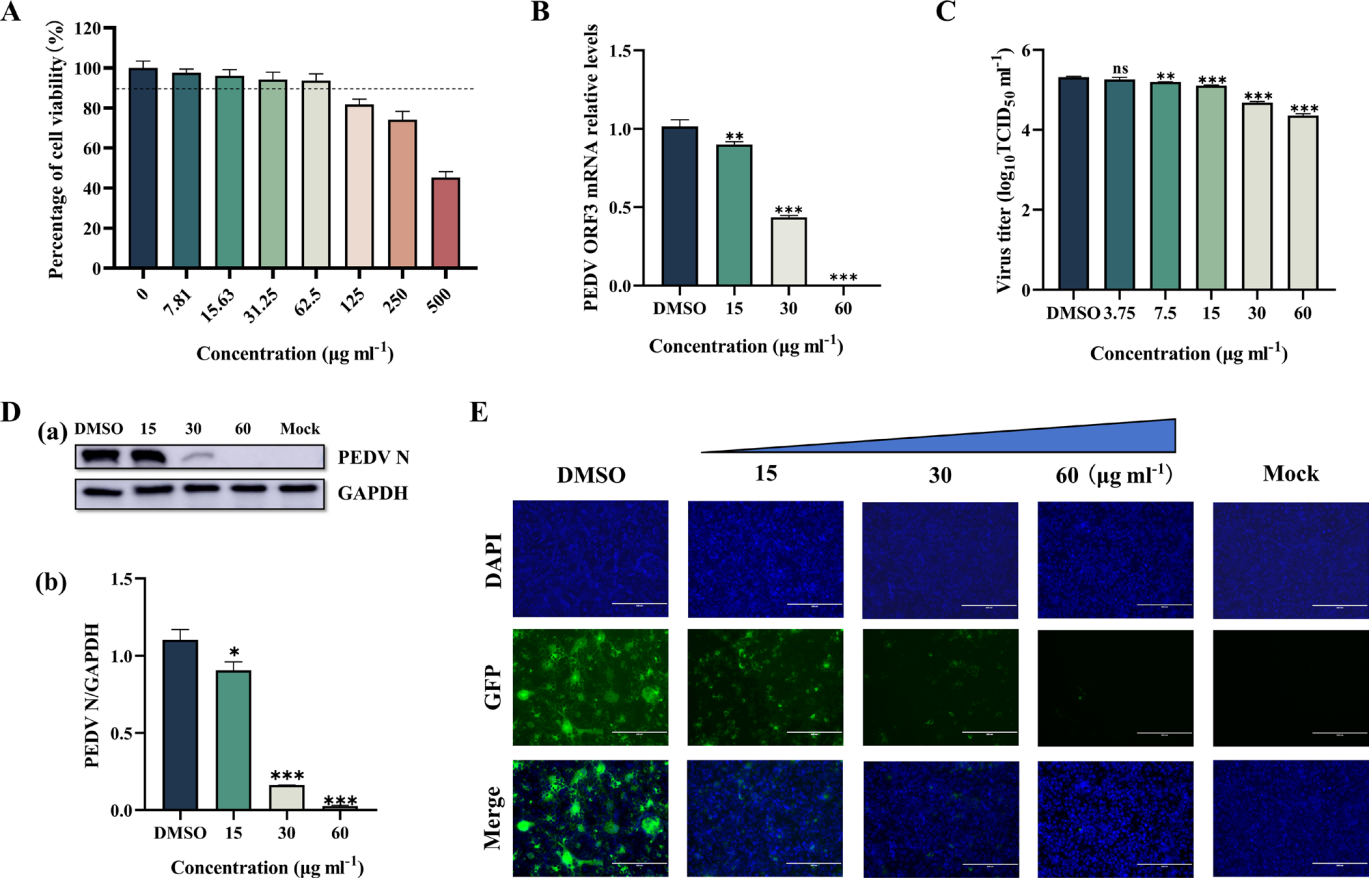


To determine the potential cytotoxicity of Le, a CCK-8 kit was used to determine the cytotoxicity of Le at concentrations of 500, 250, 125, 62.5, 31.25, 15.63, 7.82 and 3.91 µg ml^−1^ in Vero E6 cells. After treating the cells with Le at 7.81–62.5 µg ml^−1^, there was no significant change in morphology and viability remained above 90 % (Fig. 1A).

To determine the effect of Le on PEDV replication, we treated Vero E6 cells with different concentrations of Le and then infected the cells with PEDV. At 48 hpi, we detected ORF3 mRNA, TCID_50_, N protein, and M protein levels of PEDV in the cells. The PEDV ORF3 gene level was dose-dependently reduced in the Le-treated group compared with the DMSO group (the value of DMSO group is 1, 11.18 % reduction at 15 µg ml^−1^ Le, *P*<0.01; 57.15 % reduction at 30 µg ml^−1^ Le, *P*<0.001; and 99.70 % reduction at 60 µg ml^−1^ Le, *P*<0.001) (Fig. 1B). The TCID_50_ assay results showed that the viral titre of PEDV in the DMSO group cells (5.32±0.02 log_10_ TCID_50_ ml^−1^) was significantly higher than that in the Le-treated group (5.20±0.01 log_10_ TCID_50_ ml^−1^ at 7.5 µg ml^−1^ Le, *P*<0.01; 5.11±0.01 log_10_ TCID_50_ ml^−1^ at 15 µg ml^−1^ Le, *P*<0.001; 4.68±0.02 log_10_ TCID_50_ ml^−1^ at 30 µg ml^−1^ Le, *P*<0.001; and 4.36±0.04 log_10_ TCID_50_ ml^−1^ at 60 µg ml^−1^ Le, *P*<0.001) (Fig. 1C). Western blotting showed a significant reduction in N protein levels in the Le-treated group relative to the DMSO group (the value of DMSO group is 1.10, 18.01 % reduction at 15 µg ml^−1^ Le, *P*<0.05; 85.12 % reduction at 30 µg ml^−1^ Le, *P*<0.001; and 97.64 % reduction at 60 µg ml^−1^ Le, *P*<0.001) (Fig. 1D). Consistent with the above results, the PEDV-specific immunofluorescence of infected Vero E6 cells gradually diminished with increasing Le concentration, and almost no green fluorescence was seen when the Le concentration was 60 µg ml^−1^ (Fig. 1E).

**Fig. 2.**
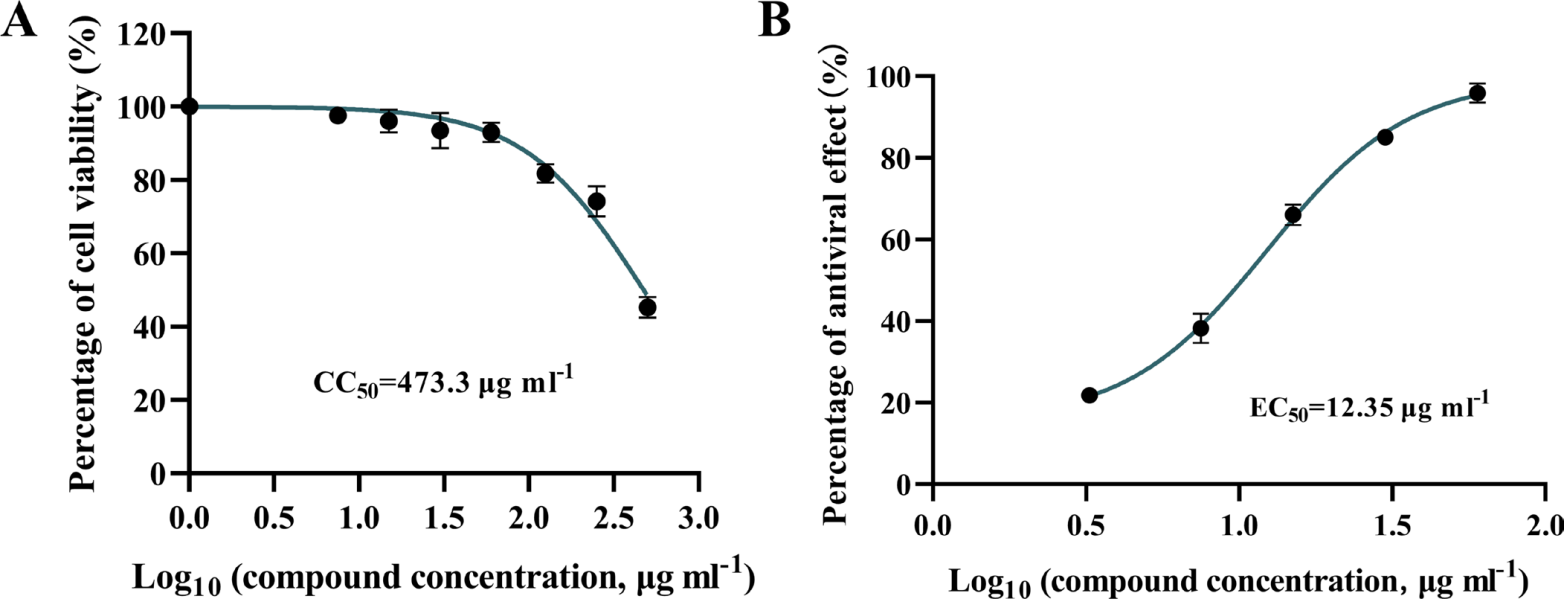
Determination of Le selection index. (**A**) CC_50_ of Le. (**B**) EC_50_ of Le.

We measured the CC_50_ of Le as 473.3 µg ml^−1^ and the 50 % effective concentration (EC_50_) as 12.35 µg ml^−1^ using the above results (Fig. 2A, B). The selectivity index (SI) of Le was calculated as 38.32 based on CC_50_/EC_50_. The above results suggested that Le had the potential to be developed as an anti-PEDV drug and inhibited PEDV replication *in vitro* in a dose-dependent manner.

### Le inhibits attachment, internalization, and replication of PEDV

**Fig. 3.**
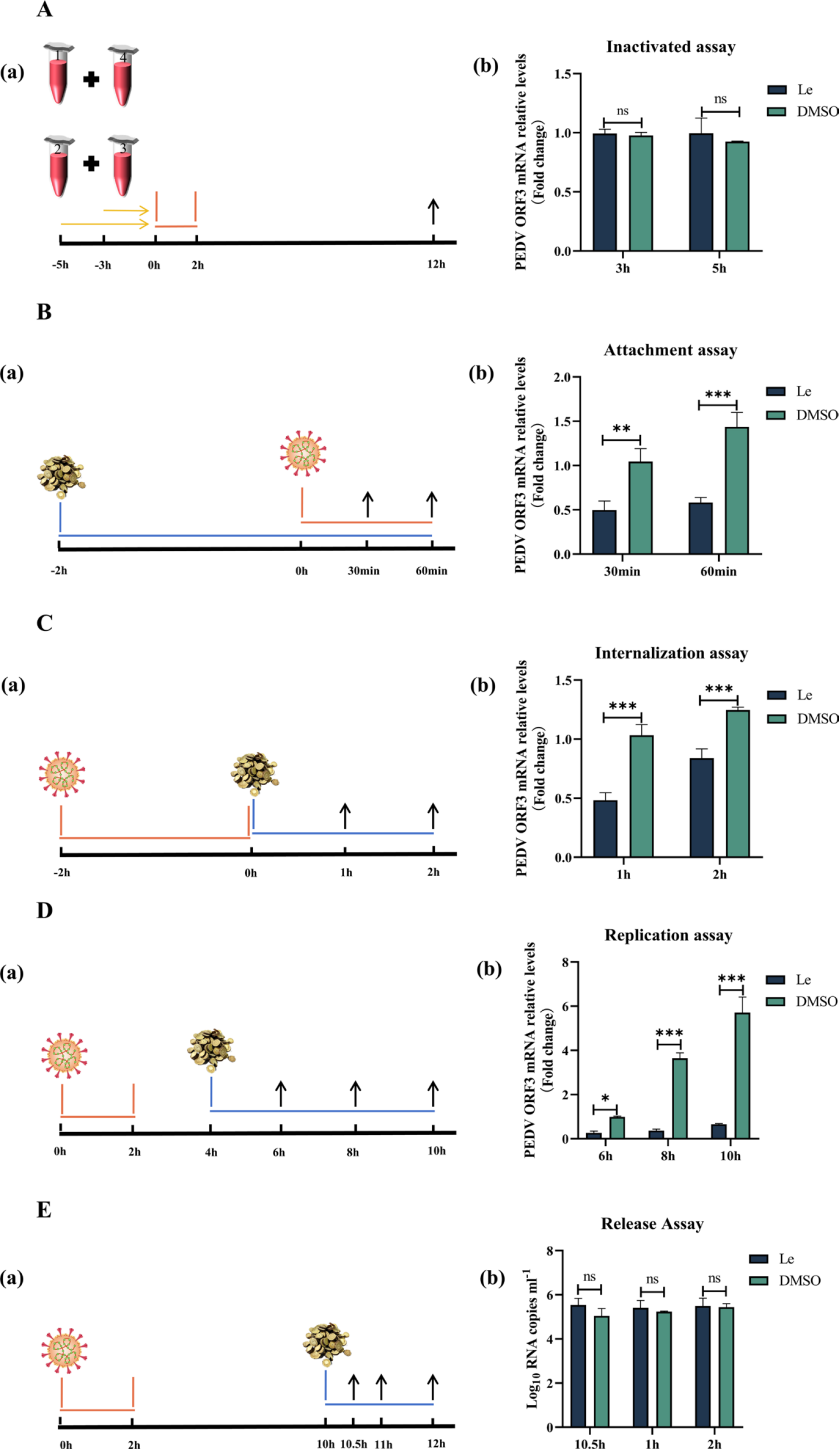
Effect of Le on inactivation, attachment, internalization, replication, and release of PEDV. (**A**) Inactivation of PEDV. Four groups: (1). PEDV +Le (acting concentration 60 µg ml^−1^); (2). PEDV +DMSO; (3). Le (acting concentration 60 µg ml^−1^); and (4). DMSO were prepared and incubated at 37 °C for 3 and 5 h. Group one was mixed with group four and group two with group three, and the mixtures were added to Vero E6 cells inoculated in six-well plates. After incubation for a further 2 h at 37 °C, the culture supernatant was replaced with fresh DMEM and incubated for a further 10 h. Cells were washed with PBS, and mRNA levels of PEDV ORF3 were measured in the cells using qRT-PCR. (**B**) Virus attachment assay. (**C**) Virus internalization assay. (**D**) Virus replication assay. (**E**) Virus release assay.

To investigate further the effect of Le on the life cycle of PEDV, PEDV ORF3 mRNA was detected by qRT-RCR. Compared with the DMSO group, Le did not kill the virus directly after co-incubation with PEDV, and the prolonged action time (5 h) did not affect the viral activity (*P*>0.05) (Fig. 3A). Treatment of Vero E6 cells with Le before PEDV infection significantly prevented viral attachment to the cells (Fig. 3B). Compared with DMSO group, PEDV ORF3 mRNA in the Le-treated group decreased by 51.92 % at 30 min after PEDV infection (*P*<0.01), and by 59.44 % at 60 min (*P*<0.001). PEDV ORF3 mRNA levels were significantly reduced at 1 h (53.51 % reduction) and 2 h (32.80 % reduction) after Le treatment compared with DMSO group, indicating that Le inhibited PEDV internalization (*P*<0.001) (Fig. 3C). Replication assays showed that Le reduced PEDV ORF3 mRNA levels by 89.76 % (*P*<0.05) at 6 h, 89.84 % (*P*<0.001) at 8 h, and 88.48 % (*P*<0.001) at 10 h compared with DMSO group (Fig. 3D). The release assay showed that there was no significant difference in the PEDV ORF3 mRNA level in the supernatant of Le-treated cells compared with the DMSO group (Fig. 3E, *P*>0.05). These results indicated that Le primarily exerted its anti-PEDV effect by influencing virus attachment, internalization, and replication.

### Le has no apparent toxicity *in vivo*

**Fig. 4.**
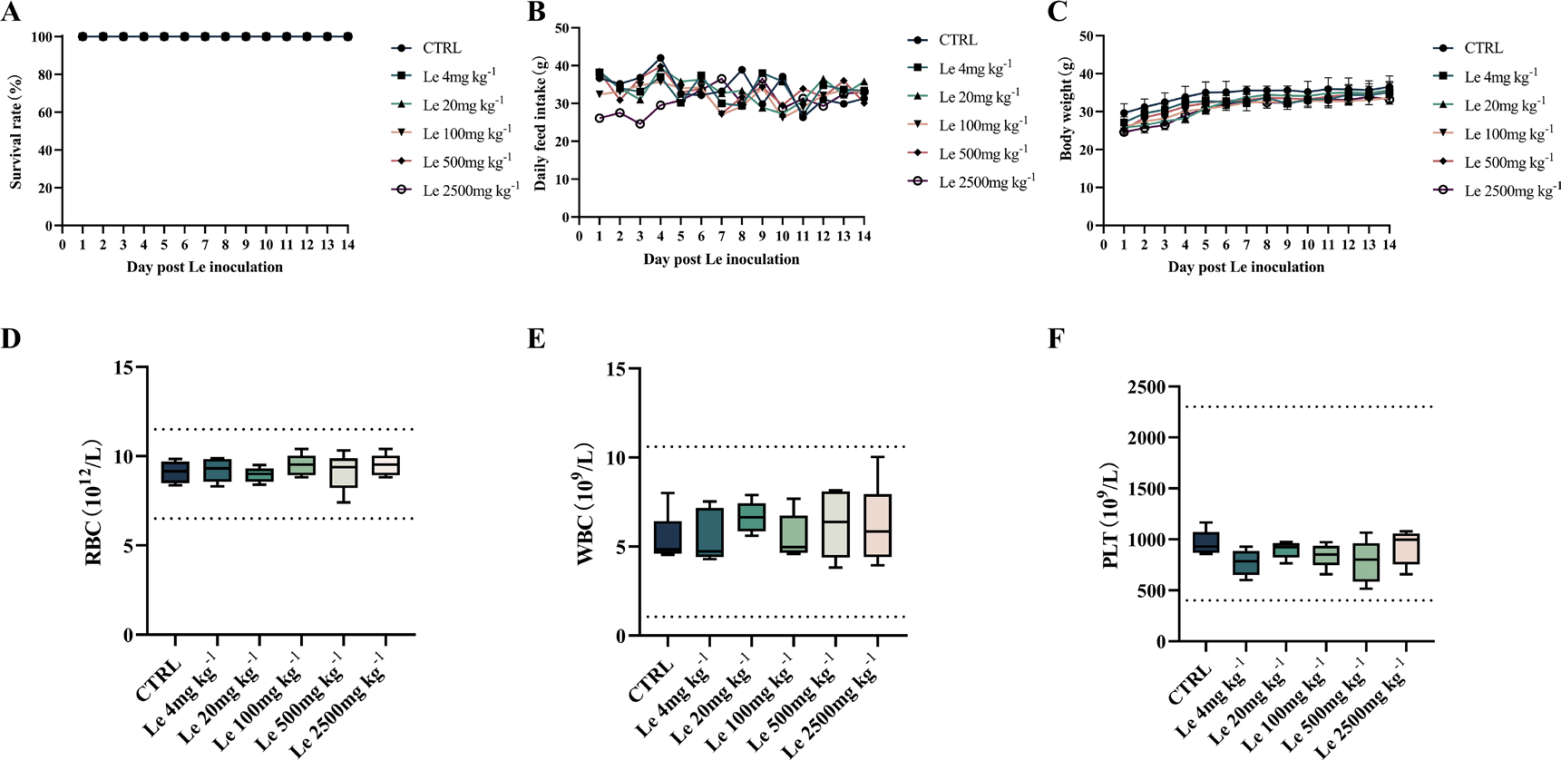
Toxicity of Le *in vivo*. (**A**) Survival curves of mice orally administered Le at a single dose of 0, 4, 20, 100, 500, or 2500 mg kg^−1^ body weight for 14 days. The subchronic toxicity evaluation of oral Le with a single dose of 0, 4, 20, 100, 500, or 2500 mg kg^−1^ in mice. (**B**) Detection of food intake and plotting of food intake change curves. (**C**) Body weight was monitored and plotted for body weight changes. Mice were killed on day 14 and assayed for (**D**) RBCs, (**E**) WBCs, and (**F**) PLTs.

To determine the safe oral dose of Le, we conducted an acute toxicity study. Mice were orally administered Le at a single dose of 0, 10, 50, 250, 500 or 2500 mg kg^−1^. Within 14 days, no deaths was observed in any treatment group (Fig. 4A). However, after oral administration of the 2500 mg kg^−1^ dose of Le, food intake of the mice was reduced in the first 4 days, whereas it was unaffected in the other treatment groups (Fig. 4B). There was no significant difference in the body weights of mice in the different treatment groups and control mice at day 14 (Fig. 4C, *P*>0.05). There was no significant effect on red blood cells (RBC), white blood cells (WBC), and platelets (PLT) in mice in all treatment groups, and the blood indices of mice were within the normal range (Fig. 4D, E, F, *P*>0.05). Therefore, the oral dose was chosen as 500 mg kg^−1^. After body surface area conversion, the oral dose to piglets was set at 125 mg kg^−1^ and was used to investigate whether Le protected piglets from PEDV infection.

### Le protects piglets against PEDV infection *in vivo*

**Fig. 5.**
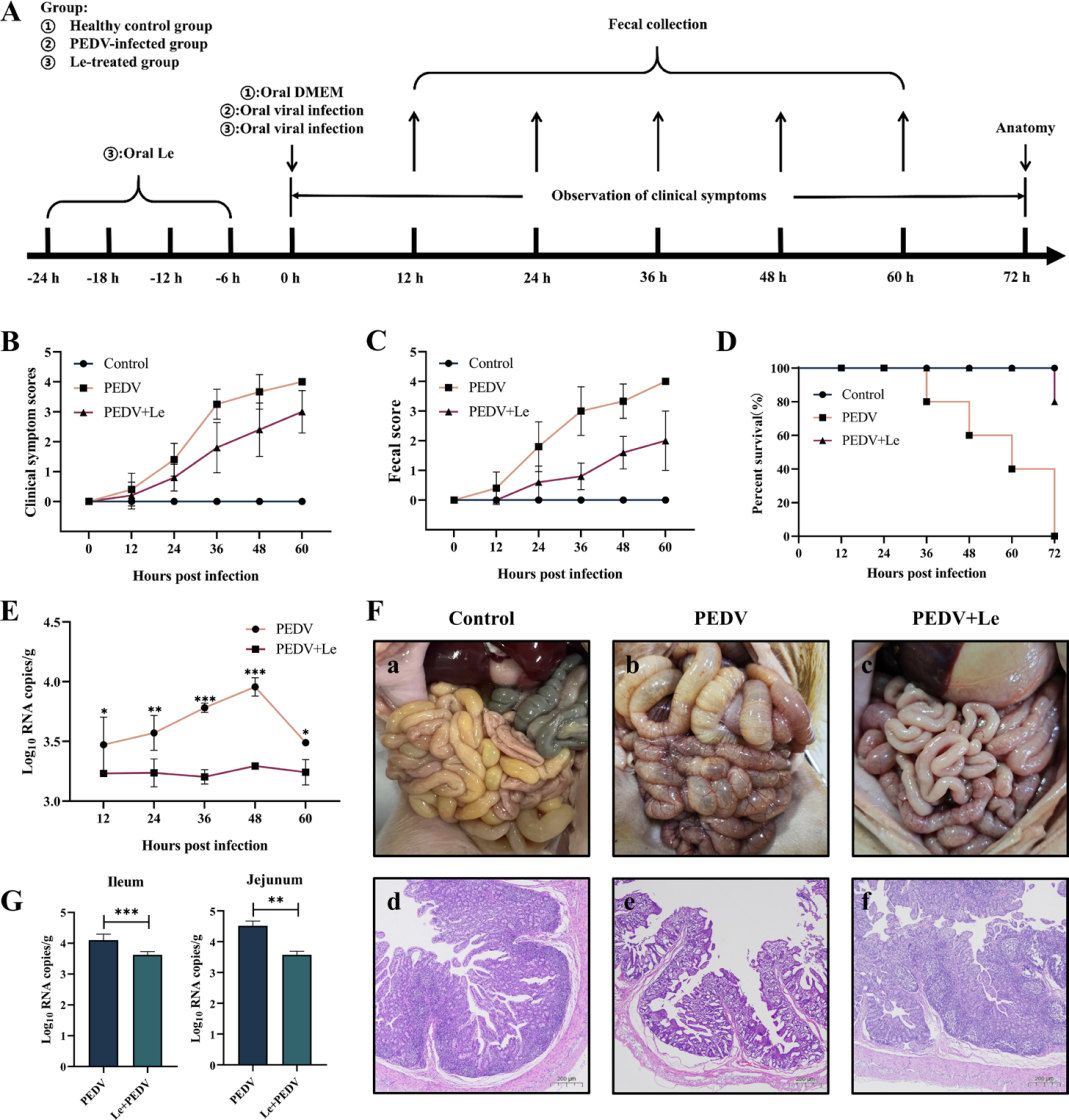
Oral administration of Le protected piglets against PEDV infection in vivo. (A) Schematic diagram of piglet experiment. (B) Clinical symptom scores of piglets in each group. (C) Faecal scores of piglets in each group. (D) Survival rate of piglets in each group. (E) Detection of faecal virus shedding. (F) Necropsy and histopathology of the intestines of PEDV-infected piglets treated with Le at 72 hpi. Macroscopic pictures of the intestines of the Control, PEDV, and PEDV +Le groups at 72 hpi (a–c). HE-stained of small intestinal tissue sections of piglets in the Control, PEDV, and PEDV +Le groups at 72 hpi (d–f). (G) The distribution of viruses in the jejunum and ileum by qRT-PCR.

Survival and clinical signs of piglets were observed daily from the time of PEDV infection, and piglet faeces were collected until all the piglets in the PEDV-infected group had died. The piglets in the PEDV-infected group developed significant diarrhoea after infection, accompanied by vomiting, loss of appetite, lethargy, rough hair, and respiratory distress. All piglets in this group died successively at 36–72 h after PEDV infection. In comparison, piglets in the Le-treated group had milder severity of clinical signs and diarrhoea after infection with PEDV (Fig. 5B, C). At 72 hpi, the survival rate of piglets in the Le-treated group was 80 % (Fig. 5D). Healthy control piglets showed no obvious clinical signs during the process and all of them survived. We used qRT-PCR to examine the levels of PEDV in collected piglet faeces. At 12–60 hpi, viral excretion in the faeces of piglets in the Le-treated group (3.23±0.02, 3.17±0.12, 3.16±0.06, 3.29±0.03 and 3.24±0.11 log_10_ RNA copies/g at 12, 24, 36 and 60 hpi, respectively) was lower than that in the PEDV-infected group (3.45±0.23, 3.57±0.15, 3.75±0.04, 3.96±0.08 and 3.49±0.03 log_10_ RNA copies/g at 12, 24, 36 and 60 hpi, respectively) (Fig. 5E).

During autopsy of the PEDV-infected group of piglets, we found severe lesions throughout the intestines. The small intestine was transparent, distended, congested, and full of yellow fluid(Fig. 5F(b)), but these symptoms were alleviated in the Le-treated group(Fig. 5F(c)). These pathologic changes were not observed in the healthy control group(Fig. 5F(a)). Similarly, histopathological analysis showed severe damage to the small intestinal villi and thinning of the lamina propria of the mucosa in the PEDV-infected piglets (Fig. 5F(e)). Small intestinal villous damage was milder in the Le-treated piglets compared with the PEDV-infected group (Fig. 5F(d)). The distribution of virus in the jejunum (3.57±0.12 log_10_ RNA copies/g) and ileum (3.62±0.11 log_10_ RNA copies/g) of the Le-treated group was lower than that in the PEDV-infected group in the jejunum (4.52±0.16 log_10_ RNA copies/g, *P*<0.01) and ileum (4.10±0.19 log_10_ RNA copies/g, *P*<0.001)(Fig. 5G). The above results suggested that Le had a protective effect against PEDV infection in piglets.

## Discussion

The emergence of mutant strains of PEDV has caused enormous problems in the swine industry. PEDV infects pig intestinal epithelial cells and causes intestinal diseases in all age groups, and piglets usually die after infection [42]. The most effective measure currently available is vaccination, but because of the ongoing mutation of PEDV, there might be variations between the vaccine strain and the prevailing strain, causing the vaccine to not offer full protection against different PEDV variant strains [43,44]. Therefore, there is an urgent need to develop effective antiviral drugs to assist the vaccine. Many studies have shown that TCM has been used as a potential source for developing new drugs for treating viral infections due to their properties, such as few adverse effects, high availability, and low cost [45,46]. Several natural constituents of TCM, including tomatidine, octyl gallate, buddlejasaponin IVb, and quercetin, have been shown to possess anti-PEDV activity [2,10, 40, 47]. Similarly, many extracts of TCM with anti-PEDV effects, such as Aloe extract, chestnut inner shell extract, and *Hypericum japonicum* extract, have been reported in previous studies [14,15, 48]. These reports confirm the feasibility of finding anti-PEDV drugs from TCM. In this study, we extracted licorice, an essential herbal medicine in TCM, and determined Le in inhibition of PEDV *in vitro* and *in vivo*.

*In vitro*, Le inhibited the PEDV HM2017 strain replication in Vero E6 cells. When the Le concentration was 60 µg ml^−1^, PEDV N protein, ORF3 mRNA, and M protein were almost completely inhibited. *Hypericum japonicum* extract and Aloe extract exerted similar effects, which may be related to the multi-component and multi-target properties of herbal extracts. This property reduces the likelihood that the virus will develop drug resistance and extends the duration of its use [14,48]. The SI of a drug can be used to determine the safety range of the drug effect, which is greater than 1.00 proving that the drug is effective. The greater the value, the greater the safety range [10]. The SI values of the bis-benzylisoquinoline substances cepharanthine, tetrandrine, and fangchinoline were reported to be 11.83, 7.08, and 4.51, respectively [49]. The SI for buddlejasaponin IVb was 12.18 [40]. The expression of PEDV N protein was almost completely inhibited by these four substances at the maximum nontoxic dose, which is similar to the antiviral effect of Le. The alkaloid tomatidine extracted from tomatoes had an SI of 13.25 [10]. The SI for ergosterol peroxide was 15.95 [50]. The expression of PEDV N protein was not completely inhibited at the maximum nontoxic dose of these two substances. Le had an SI of 38.32, which demonstrated a greater safety threshold. This may be related to the fact that multiple components in Le exert anti-PEDV effects [35,51].

The diverse composition of TCM extracts means that the pathways by which they exert their antiviral effects are complex [52]. Le acted mainly on the attachment, internalization, and replication stages of PEDV. Other different TCM extracts and natural compounds also exert inhibitory effects at different stages of the PEDV life cycle. Some studies have shown that epigallocatechin gallate acts as a viral inhibitor throughout the viral life cycle [9]. Aloe extract not only has the effect of directly inactivating the virus but also plays a role in the later stages of the virus lifecycle [14]. Chestnut inner shell extract plays a role in the early stages of PEDV infection [15]. Similarly, some active ingredients contained in licorice exert antiviral effects at different stages of the viral life cycle, such as glycyrrhizin, glycyrrhetinic acid, and licorice-saponin A3, which may underlie the anti-PEDV effects exerted by Le. Glycyrrhizin prevents SARS-CoV-2 from entering cells by targeting angiotensin-converting enzyme 2 [53]. Licorice-saponin A3 may inhibit viral entry into cells by targeting nsp7 [35]. Glycyrrhetinic acid binds to the spike protein receptor binding domain of SARS-CoV-2 and acts as an antiviral during the attachment phase [51]. Glycyrrhizin, glycyrrhetinic, licochalcone, and glabridin are potential inhibitors of SARS-CoV-2 3CLpro and inhibit viral replication. Le may exert its anti-PEDV effects through similar pathways [51,54]. Le contains some components with anti-inflammatory and immunomodulatory effects, which can inhibit host inflammation and exert anti-PEDV effects through pathways such as c-Jun N-terminal kinase, mitogen-activated protein kinase, and nuclear factor-kappa B [24,35, 55, 56]. The present study was only a preliminary exploration of the pathways through which Le exerts its antiviral effects; the specifics of which need to be looked at in the future.

Safety in animals is one of the primary considerations for antiviral drug candidates [57]. Evaluation of the toxicity of a drug helps to determine the amount of the drug to be used and to minimize toxic adverse effects [58]. In this study, we validated the toxicity of Le in mice and determined the optimal dose to be administered to piglets based on relative body surface area conversion. Mice did not die after 14 days oral administration of high concentrations of Le (2500 mg kg^−1^), demonstrating that Le had a wide safety range for clinical use in animals. Subsequently, Le was evaluated for its antiviral effects *in vivo* using an established animal model of PEDV-infected piglets. The immune system of piglets is immature and cannot produce sufficient antibodies to resist infection [2]. Therefore, infection and rapid proliferation of PEDV can lead to the swift death of infected piglets. Our data showed that all piglets in the PEDV-infected group died at 72 hpi, but piglets in the Le-treated group had a survival rate of 80 %. Although the piglets in the Le-treated group still showed mild lesions after autopsy, the mortality rate was significantly lower than that in the PEDV infection group. This may have been because of the ability of Le to reduce the viral load in the intestinal tract [2,14, 40, 48]. This mechanism provides a vital survival opportunity for piglets and enhances their ability to resist PEDV. Compared with the PEDV-infected group, the survival rate of piglets treated with octyl gallate was 75%, and the clinical symptoms and intestinal damage of piglets in the treatment group were reduced [2]. The survival rate of quercetin-treated piglets was 33.3%, and the intestinal damage was less severe than the PEDV-infected piglets [47]. After the injection of 1 mg kg^−1^ buddlejasaponin IVb into PEDV-infected piglets, no significant clinical signs and intestinal damage were observed, and the therapeutic efficacy was superior to Le [40]. The positive efficacy of buddlejasaponin IVb may be related to the injectable mode of administration, which improves drug utilization. Multiple (ten) injections of the drug into piglets by the researchers also improved the therapeutic effect of buddlejasaponin IVb. Clinical signs of infected piglets were reduced after treatment with Aloe extract, and the survival rate of piglets in the treated group was 100 % when the mortality rate of piglets in the PEDV-infected group was 50 % [14]. These studies suggest that Le has relatively good therapeutic effects.

In addition to safety and efficacy, cost is also a key issue in whether a drug can be used in practice. Some reports indicate that it may take about 10 years to develop a new drug for clinical application, and the cost of consumption is hundreds of millions of dollars [17]. Such long lead times and high costs make application of new drugs to treat piglets impractical. As a result, the drug development model of new use of old drugs is increasingly recognized [59]. Organic compounds from natural sources used in the past are now also used for the treatment of various diseases; these compounds are either used in their natural form or as lead molecules for developing synthetic and semi-synthetic analogues with improved drug-forming properties [17,60, 61]. For example, hypericin, quercetin, GC376, and tomatidine, which have been reported to have anti-PEDV effects, are typical examples of new uses of old drugs [10,11, 62, 63]. However, the price of these single-ingredient drugs still far exceeds what one would expect to spend on treating piglets. For example, GC376, a synthetic drug, costs more than $100 g^−1^. Additionally, certain active ingredients found in TCM can also be costly due to the expenses associated with their extraction. Compared with these antiviral drugs, the preparation process of Le is simple; its raw material, licorice, is widely sourced, easy to obtain, and its price is low. In this study, the cost of treatment per piglet could be controlled at about $1 per piglet. The low price suggests that Le has the potential to be used on a large scale in practice.

In summary, Le showed intense anti-PEDV activity *in vitro* and *in vivo*. This is the first study on the anti-PEDV activity of Le and provided an effective strategy for the development of cheap and efficient anti-PEDV drugs.

## Abbreviations

CC_50_: 50% cytotoxic concentration
CCK-8: Cell Counting Kit-8
DMEM: Dulbecco’s Modified Eagle’s medium
DMSO: dimethyl sulfoxide
EC_50_: 50% effective concentration
hpi: hour post-inoculation
Le: licorice extract
PBS: phosphate-buffered saline
PED: porcine epidemic diarrhea
PEDV: porcine epidemic diarrhea virus
PLT: platelets
RBC: red blood cell
SI: selection index
TCID_50_: 50% tissue culture infective dose
TCM: traditional Chinese medicine
WBC: white blood cell
